# Investigation into the Relationship between Spermidine Degradation and Phenolic Compounds Biosynthesis in Barley Seedlings under Ultraviolet B Stress

**DOI:** 10.3390/plants12203533

**Published:** 2023-10-11

**Authors:** Chong Xie, Yahui Zhu, Chaoqun Leng, Qiaoe Wang, Pei Wang, Runqiang Yang

**Affiliations:** 1 College of Food Science and Technology, Whole Grain Food Engineering Research Center, Nanjing Agricultural University, Nanjing 210095, China; xiechong@njau.edu.cn (C.X.); wangpei@njau.edu.cn (P.W.); 2 College of Food Science and Technology, Tibet Agriculture and Animal Husbandry University, Linzhi 860000, China; wangqe@th.btbu.edu.cn; 3 Beijing Key Lab of Plant Resource Research and Development, Beijing Technology and Business University, Beijing 100048, China; zhuyahui@xza.edu.cn

**Keywords:** barley seedling, spermidine, phenolic compounds, gamma-aminobutyric acid

## Abstract

Barley germination under ultraviolet B (UV-B) illumination stress induces effective accumulation of phenolic compounds in the barley. Spermidine can enhance the biosynthesis of phenolic compounds and alleviate the oxidative damage caused by UV-B. To better understand the function of spermidine, inhibitors of enzymes that are involved in the degradation of spermidine and the synthesis of gamma-aminobutyric acid (GABA), the product of spermidine degradation, were applied to barley germinated under UV-B treatment. The results showed a more severe oxidative damage, and a decrease in phenolic acid contents were observed when spermidine degradation was inhibited. However, GABA application did attenuate an increase in electrolyte permeability and MDA content caused by UV-B induced oxidative damage and improved the respiration rate. Meanwhile, GABA application can elevate the accumulation of phenolic compounds by ca. 20%, by elevating the activities of some key enzymes. Furthermore, the application of GABA, together with the inhibitor of spermidine degradation, can alleviate its suppression of the synthesis of phenolic acids, and resistance to UV-B stress. In conclusion, spermidine alleviated oxidative damage and enhanced the accumulation of phenolic compounds using its degradation product.

## 1. Introduction

Phenolic compounds play important roles in plant growth, development and defense against environmental stress [1,2]. Meanwhile, these substances have various physiological activities in the human body and epidemiological studies have shown that long-term consumption of foods rich in phenolic compounds may provide protection against many chronic diseases [3,4]. Therefore, food products that are high in phenolic compounds have gained increased popularity.

Barley is a nutritious cereal that offers several health benefits, but, when compared to wheat and rice, it does have palatability and processing issues, which limit their uses to the beer industry. Germination is an effective way to increase the nutrient content and alter the flavor profiles of cereals and some abiotic stresses can be used to enhance the accumulation of phenolic compounds during germination [5,6,7]. UV-B (ultraviolet B light) is part of the electromagnetic spectrum that has a wavelength ranging from 280 to 320 nm; it can induce oxidative damage in grains, which leads to a higher accumulation of phenolic compounds but a significantly lower length and fresh weight.

Polyamines, including putrescine, spermidine and spermine, have diverse roles in cellular processes in plants, such as apoptosis, cell proliferation, DNA synthesis, gene expression and signal transduction [8]. In our previous study, exogenous spermidine not only enhanced the accumulation of phenolic compounds, but also alleviated the oxidative damage caused by UV-B radiation [9]. In plants, polyamine degradation is catalyzed by polyamine oxidase and renders aminobutyraldehyde, which is subsequently converted into gamma-aminobutyric acid (GABA) by the action of aminoaldehyde dehydrogenase (AMADH) [10]. GABA, a four-carbon molecule, plays various roles in plants, including resistance to both abiotic and biotic stress [11]. GABA has been shown to influence the biosynthesis of phenolic compounds and antioxidant systems in soybean sprouts germinated under NaCl stress [7].

To better understand the role of spermidine in phenolic compound biosynthesis and the establishment of antioxidant adaptive responses of barley during germination, whether the effect of spermidine is directly from the action of itself or by its degradation product, i.e., GABA, should be clarified. Therefore, in the present study, barley seedlings germinated under UV-B stress were treated with GABA (G seedling), aminoguanidine hydrochloride (AG), the inhibitor of polyamine oxidase (A seedling), 1-ethyl-(3-dimethylaminopropyl), carbon diimide hydrochloride (EDC), the inhibitor of AMADH (E seedling) and EDC together with GABA (EG seedling), respectively. The growth, content of phenolic compounds and antioxidant capacity of seedlings with different treatments were investigated.

## 2. Results

### 2.1. Growth of Barley Seedlings

**Figure 1:**
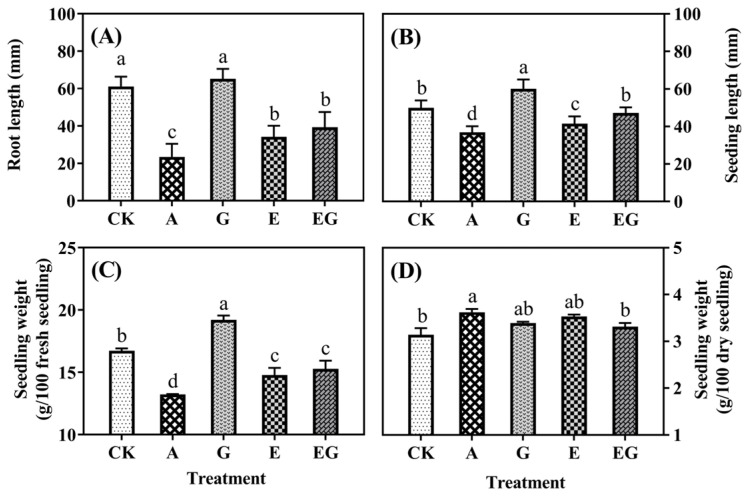
Root length (**A**), seedling length (**B**), fresh weight (**C**) and dry weight (**D**) of barley seedlings. CK are seedlings treated with distilled water. A, G, E and EG are seedlings treated with aminoguanidine hydrochloride, gamma-aminobutyric acid (GABA), 1-ethyl-(3-dimethylaminopropyl) carbon diimide hydrochloride (EDC) and GABA together with EDC, respectively. Different lowercase letters (a–d) represent significant difference among treatments (n = 3; *p* < 0.05).

As shown in Figure 1A,B, treatments with both AG and EDC resulted in significantly (*p* < 0.05) shorter lengths of seedling and root. Application of GABA led to a 20% increase in seedling length. The barley seedling treated with GABA and EDC together exhibited a significantly (*p* < 0.05) longer seedling length (ca. 47 mm) than E seedling. The CK seedling had ca. 17 g/100 seedlings of fresh weight and ca. 3.1 g/100 seedlings of dry weight (Figure 1C,D). The highest fresh weight of seedlings was found in the G seedling, which was approximately 19 g/100 fresh seedlings, and the lowest fresh weight was observed in the A seedling (13 g/100 fresh seedlings). However, the A seedling had the highest level of dry weight (3.62 g/100 fresh seedling) and the lowest level of dry weight (3.14 g/100 fresh seedling) was found in the CK seedling.

### 2.2. Content of Malondialdehyde, Electrolyte Leakage, Respiratory Rate and Spermidine Content

**Figure 2:**
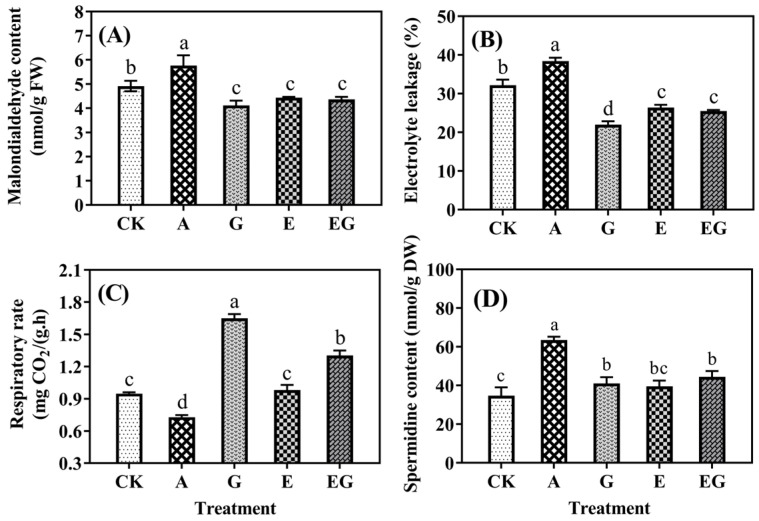
Content of malondialdehyde (**A**), electrolyte leakage (**B**), respiratory rate (**C**) and contents of spermidine (**D**) in barley seedlings (n = 3). Different lowercase letters (a–d) represent significant difference among treatments (n = 3; *p* < 0.05).

As shown in Figure 2A–C, the application of AG resulted in a significantly (*p* < 0.05) higher level of MDA content (5.76 nmol/g FW) and electrolyte leakage (38%) than the CK seedling. Both GABA and EDC can significantly (*p* < 0.05) decrease the MDA content and electrolyte leakage. The highest and lowest respiratory rates were found in the G and A seedlings, respectively, and EG seedling had a ca. 40% higher respiratory rate than E seedling.

The content of spermidine in the CK seedling was ca. 35 nmol/g DW (Figure 2D). Application of AG and GABA significantly (*p* < 0.05) increased the content of spermidine by 82.79% and 18.38%, respectively. EDC had no significant (*p* > 0.05) effect on spermidine content but a significantly (*p* < 0.05) higher spermidine content was observed when it was applied in combination with GABA.

### 2.3. Content of Phenolic Compounds and Phenolic Acids

**Figure 3:**
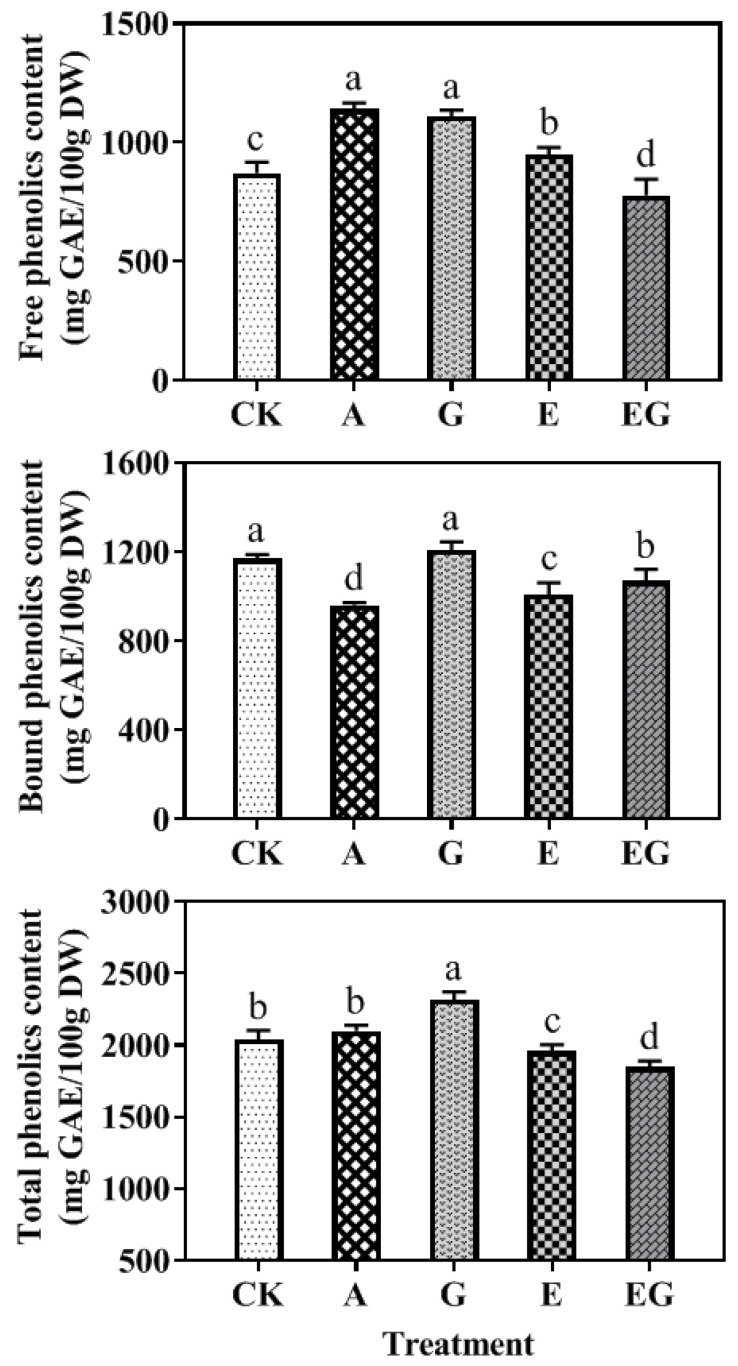
Content of free, bound and total phenolic compounds in barley seedlings (n = 3). Different lowercase letters (a–d) represent significant difference among different treatments (*p* < 0.05).

As shown in Figure 3, the A seedling had a significantly (*p* < 0.05) higher level of free phenolic compounds (1140 mg GAE/g DW) and a lower level of bound phenolic compounds (ca. 960 mg GAE/g DW) than the CK seedling. The addition of GABA resulted in a significant (*p* < 0.05) increase in the content of both free phenolic compounds (increased by 21.18%) and total phenolic compounds (increased by 13.42%) when compared to the control. EDC treatment also led to a significant (*p* < 0.05) increase in free phenolic compounds and decrease in bound phenolic compounds. However, the content of total phenolic compounds in E seedling (1958 mg GAE/g DW) was significantly (*p* < 0.05) lower than the control seedling (2043 mg GAE/g DW). The addition of GABA to EDC resulted in the lowest level of free (778 mg GAE/g DW) and total phenolic compounds (1849 mg GAE/g DW).

As shown in Table 1, ferulic acid exhibited the highest content among all the detected phenolic acids in all the seedlings, followed by *p*-coumaric acid, sinapic acid and caffeic acid. Among them, ferulic acid and *p*-coumaric acid were mainly present in the bound forms but caffeic acid was mainly present in the free form. The contents of vanillic acid, *p*-hydroxybenzoic acid, protocatechuic acid and syringic acid were present in relatively low levels. After germination, the content of total ferulic acid decreased by 19.49% under AG treatment but increased by 29.80% when GABA was added under UV-B stress. A 20.16% decrease in total ferulic acid content was observed by the addition of EDC and this inhibitory effect of EDC was alleviated by exogenous GABA. A similar trend was observed in other types of phenolic acids, such as *p*-hydroxybenzoic acid, vanillic acid and sinapic acid. Free protocatechuic acid was not detected under EDC treatment, and free syringic acid was only detected under UV-B stress with the addition of GABA.

### 2.4. Activities of Key Enzymes Related to Synthesis of Phenolic Acid

**Figure 4:**
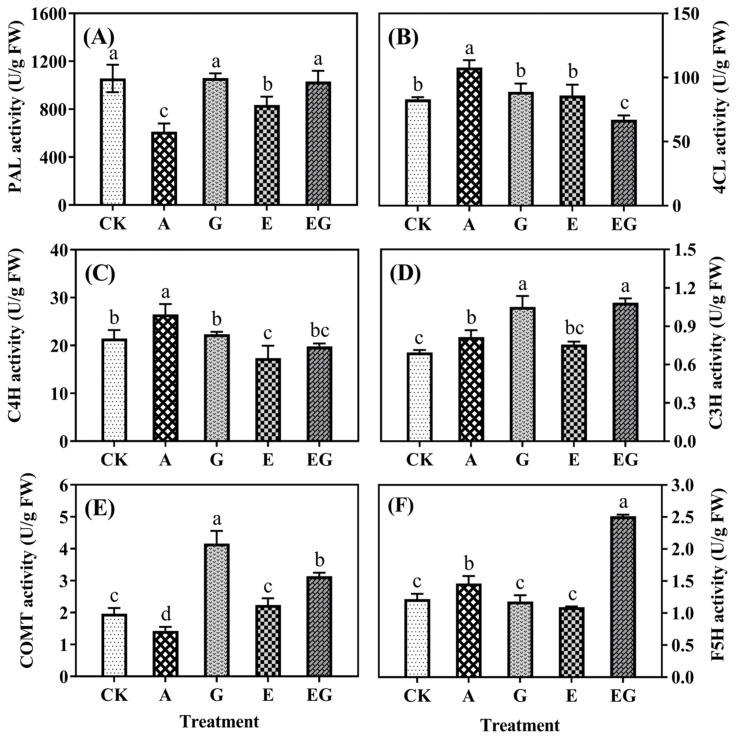
Activities of PAL (**A**), 4CL (**B**), C4H (**C**), C3H (**D**), COMT (**E**) and F5H (**F**) in barley seedlings (n = 3). Different lowercase letters (a–d) represent significant difference among treatments (*p* < 0.05).

Under UV-B stress, AG treatment significantly (*p* < 0.05) reduced the activities of phenylalanine ammonia lyase (PAL) and caffeic acid-o-methyltransferase (COMT) but increased the activities of 4-coumarate coenzyme A ligase (4CL), coumarate 3-hydroxylase (C3H), cinnamic acid 4-hydroxylase (C4H) and ferulate 5-hydroxylase (F5H), as shown in Figure 4. The addition of GABA significantly (*p* < 0.05) increased the activities of C3H and COMT by 52.17% and 111.17%, respectively, but had no significant (*p* > 0.05) influence on the activities of other enzymes. Meanwhile, the highest activities of PAL and COMT were found in the G seedling. EDC treatment decreased the activities of PAL and C4H by 20.99% and 19.14%, respectively, and the lowest activities of C4H and F5H were found in the E seedling. The EG seedling had significantly (*p* < 0.05) higher activities of PAL, C3H, COMT and F5H than the E seedling, and the highest activities of C3H and F5H were observed in the EG seedling.

### 2.5. Antioxidant Capacity

As shown in Table 2, total ABTS radical scavenging in all seedlings ranged from 77.06 μmol TE/g DW (EG seedling) to 83.80 μmol TE/g DW (A seedling). In the A and G seedlings, free phenolic compounds had a higher contribution than the bound forms. Total DPPH radical scavenging in all seedlings ranged from 75.79 μmol TE/g DW (A seedling) to 43.53 μmol TE/g DW (G seedling) and bound phenolic compounds had a higher contribution than the free forms in all seedlings.

## 3. Discussion

Polyamines are involved in various biological processes in plants, such as growth, development and adaption to environmental stresses [12]. The previous study showed the application of exogenous spermidine increased the length and fresh weight of barley seedling under UV-B stress [9]. In the present study, the addition of either AG or EDC significantly (*p* < 0.05) elevated the content of endogenous spermidine through the inhibition on polyamine oxidase or AMADH (Figure 2D). However, significant (*p* < 0.05) decreases in the length and fresh weight of barley seedlings under UV-B stress after treatments of AG or EDC suggested the degradation product of spermidine, i.e., GABA, may have a role in the adaption of the germinating barley seed to abiotic stress, as this has been shown to occur in other plants [13,14,15].

GABA is involved in the regulation of various physiological plant responses, such as stress tolerance, growth regulation and signal transduction [16]. The results of the present study showed that GABA enhanced the growth of barley seedlings under UV-B stress by mitigating the increase in electrolyte permeability and MDA content caused by oxidative damage (Figure 2). Additionally, GABA promoted the respiration rate, which has been shown to positively correlate with seedling growth [17]. Meanwhile, exogenous GABA also increased the content of spermidine, which can be attributed to its suppression on polyamine oxidase as reported in the literature [18].

Phenolic compounds are important secondary metabolites ubiquitously present in plants, and their biosynthesis primarily begins with the conversion of the amino acid phenylalanine to cinnamic acid by the action of PAL [19]. Then, cinnamic acid undergoes hydroxylation through the catalyzation of C4H to form *p*-coumaric acid, which serves as a precursor for other phenolic acids generated via enzymatic reactions facilitated by 4CL, C3H, COMT and F5H [20]. Our previous study showed exogenous spermidine enhanced the biosynthesis of phenolic acids in barley during germination through elevating the activities of these enzymes and an opposite trend was observed when the inhibitor of endogenous spermidine synthesis was applied [9].

**Figure 5:**
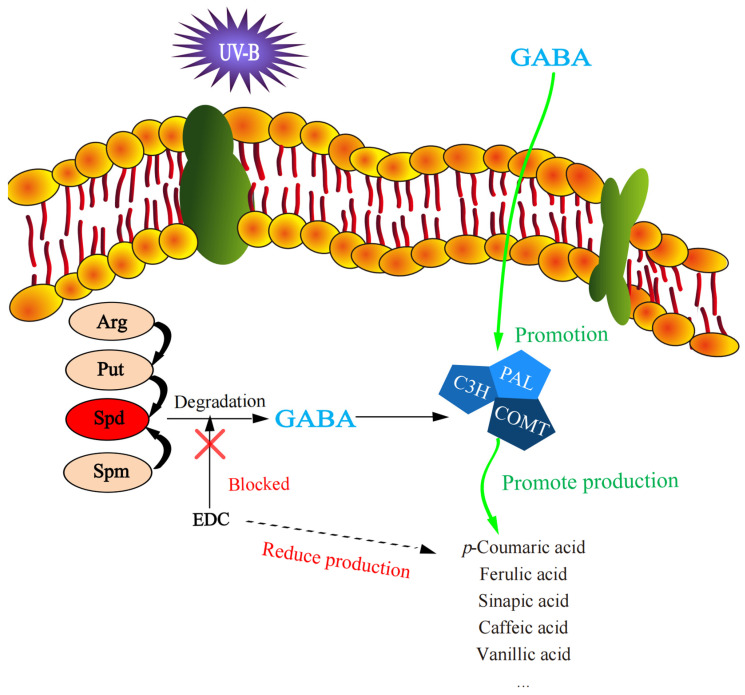
The speculative schema of the phenolic compound synthesis. Arg, Put, Spd and Spm were arginine, putrescine, spermidine and spermine, respectively.

In the present study, although the content of endogenous spermidine was significantly (*p* < 0.05) increased when AG or EDC was applied, the content of phenolic acids, such as ferulic acid and *p*-coumaric acid were notably reduced (Table 1) even though total phenolic content did not significantly (*p* > 0.05) differ from that of the control seedling (Figure 1). The reduction in phenolic acid biosynthesis can be explained by the inhibition of the activity of PAL, the pivotal enzyme in phenolics biosynthesis [21]. Furthermore, it was shown that the application of exogeneous GABA can alleviate the suppression of EDC in the synthesis of phenolic acids. Taken together, these results suggest that the effect of spermidine on biosynthesis of phenolic acid in barley germinated under UV-B stress may mainly result from its degradation product (see Figure 5 below).

Phenolic acids in cereals exist in both free and bound forms, and free phenolic acids are generally located in the outer layer of the grains, while bound phenolic acids are covalently bound with structural molecules, such as celluloses, hemicelluloses and proteins [22]. In barley grains, the cell wall polysaccharides, such as arabinoxylans, can interact with phenolic acids, resulting in their retarded digestibility in the upper part of gastrointestinal tract [23]. Interestingly, treatments of AG and EDC resulted in the conversion of bound phenolic compounds and certain phenolic acids to the free forms even though their contents were not increased. In the seedlings treated with GABA, the contents of phenolic compounds or phenolic acids were mainly increased in the bound forms.

## 4. Materials and Methods

### 4.1. Materials and Reagents

Barley grains (cultivar: Su Hullless 2) provided by the Jiangsu Academy of Agricultural Sciences were harvested in Jiangsu Province, China, in 2020 and were stored at −20 °C before use. All the chemicals were purchased from Sigma-Aldrich (St. Louis, MO, USA).

### 4.2. Experimental Design

The barley grains were immersed in a sodium hypochlorite solution (0.5% *v*/*v*, 15 min) after rinsing with deionized water. Then, the grains were rinsed with distilled water until they attained a neutral pH. After soaking for 6 h in deionized water at 25 °C, barley grains were evenly distributed in a dark incubator maintained at 25 °C to initiate the germination process. Following a one-day germination without light, grains underwent exposure to UV-B (intensity was 38 μW/cm^2^) for a duration of 6 h every 24 h. Throughout the duration of the experiment, the culture solutions were changed at intervals of 8 h. Five distinct types of solution were utilized, which included distilled water as the control (CK), a solution of 2.5 mM AG (A), a solution of 5 mM GABA (G), a solution of 0.5 mM EDC (E) and a combination of 0.5 mM EDC and 5 mM GABA (EG). On the fourth day of germination, a portion of the barley seedlings were randomly selected for measurement as a fresh sample and the remaining seedlings were stored at −80 °C after being rinsed with deionized water.

### 4.3. Determination of Average Seedling Length and Weight, MDA Content, Electrolyte Leakage and Respiratory Rate

The lengths and weights of seedlings were measured by vernier caliper and balance, respectively, as reported in the previous study [9].

The MDA content, electrolyte leakage and respiratory rate were determined as described in the previous study [15]. For determination of MDA content, 10 fresh seedlings were mixed with 5 mL 10% trichloroacetic acid solution after grinding and centrifuged (10,000× *g*, 20 min). Then, 2 mL of the supernatant was combined with 2 mL of 0.67% thiobarbituric acid. After boiling for 30 min, the mixtures were centrifuged and the supernatants (4000× *g*, 15 min) were used for measurement of absorbances (450 nm, 532 nm and 600 nm). The MDA content was calculated as c = 6.45 × (A532 − A600) − 0.56 × A450.

For determination of electrolyte leakage, approximately 1 g of fresh seedlings that had been cut into lengths of 3 mm were immersed in 30 mL distilled deionized water. The electrolyte leakage was calculated based on the change in conductivity before and after the boiling water bath for 10 min. Respiratory rate was determined by the CO_2_ production from 5 g of barley seedlings in a jar during 30 min, and expressed as mg CO_2_/(g·h).

### 4.4. Determination of Spermidine Content

To determine the content of spermidine, approximately 1 g of barley seedlings were ground in an ice bath after being added to 4 mL of pre-cooled 5% HClO4 (*v*/*v*) and placed in the ice bath for 1 h. Subsequently, the mixture was centrifuged (15,000× *g*) for 20 min at 4 °C and supernatant was utilized for the determination of spermidine by the HPLC method according to Hu et al. [24]. Briefly, 1 mL supernatant was combined with 2 mL 2 M NaOH and 10 μL of benzoyl chloride. The mixture was vortexed for 30 s and then incubated in a water bath (37 °C) for 30 min. Subsequently, 2 mL of saturated NaCl solution was added to the mixture, followed by the addition of 3 mL of cold diethyl ether for the purpose of extracting benzoyl polyamide. The resulting mixture was then subjected to centrifugation (10,000× *g*, 5 min). From the resulting centrifuged mixture, 1.5 mL of the ether phase was collected and dried using nitrogen gas. To prepare the samples for analysis, 1250 μL of 64% methanol was added to the dried ether phase, which was then passed through a 0.45 μm organic phase filter membrane. The determination of both the samples and the standard solutions was performed using an Agilent 1200 Series HPLC system, equipped with an XDB C18 column (4.6 × 250 mm, 5 μm) and a SPERMIDINE-6AV UV detector operating at a wavelength of 254 nm. The mobile phase consisted of a mixture of methanol and ultrapure water in a ratio of 64:36, and the column temperature was set to 30 °C. The flow rate was maintained at 0.6 mL/min, and an injection volume of 20 μL was used.

### 4.5. Determination of the Contents of Phenolic Compounds and Phenolic Acids

Extraction and quantification of PCs were conducted according to Xie et al. [25] with slight modification. The freeze-dried samples were finely ground and sieved using a 60-mesh sieve. Approximately 1 g of the resulting powders were extracted with methanol (80%, *v*/*v*) and shaken in the absence of light for one hour under a nitrogen atmosphere at a temperature of 25 °C and a stirring speed of 200 rpm for 3 cycles. Following the shaking process, the mixture underwent centrifugation at 10,000× *g* (4 °C, 15 min). The supernatants from 3 cycles were evaporated by a rotary evaporator and the dried extracts were dissolved in 10 mL of 50% methanol (*v*/*v*) as the free phenolic compound.

The residues remaining from the extraction of the free phenolics were hydrolyzed by reaction with 2 M NaOH. The hydrolysis process occurred in a shaker (25 °C, 200 rpm) for a duration of 4 h, in the dark. The resulting hydrolysates were adjusted to an acidic pH (1.5–2.0) by 6 M HCl. Subsequently, the mixtures were extracted with ethyl acetate for 15 min followed by centrifugation at 10,000× *g* (4 °C, 5 min). The ethyl acetate layers collected from three rounds of centrifugation were evaporated in a rotary evaporator and the dried extracts were redissolved in 10 mL of 50% methanol (*v*/*v*) and protected with nitrogen gas, constituting the extract containing the bound phenolic compounds.

The contents of bound and free phenolic compounds were determined by the Folin phenol method. The contents of free and bound phenolic acids were measured by a high-performance liquid chromatography (HPLC) method (Shimadzu LC-20A, column C18 110A, 5 μm particle size, 4.6 × 150 mm). The mobile phases were water (contained 0.1% acetic acid) and methanol (contained 0.1% acetic acid) and the flow rate was set at 0.9 mL/min with a column temperature set at 35 °C. The quantification of phenolic acids was conducted by measuring their absorbance at a wavelength of 280 nm and subsequently calculating the concentrations based on standard curves.

### 4.6. Determination of Enzyme Activities

To determine the activities of enzymes related to phenolic compounds biosynthesis, ground barley seedlings were mixed with 50 mM phosphate buffer (pH 7.4), which contained 5 mM dithiothreitol, 0.1 mM ethylenediamine tetraacetic acid and 1% polyvinyl pyrrolidone. After centrifugation (15,000× *g*, 15 min), The activities of C3H, COMT and F5H in supernatants were determined by the corresponding kits that purchased from Jiancheng Bioengineering Research Institute (Nanjing, China). The activities of 4CL, PAL and C4H were determined according to the previous study [25].

### 4.7. ABTS and DPPH Radical Scavenging Activity Assay

ABTS and DPPH’s radical scavenging abilities were determined according to the literature [25] with minor modifications. Briefly, extracts containing free (50 μL) or bound (80 μL) phenolics were combined with ABTS (3.0 mL) or DPPH (3.8 mL) solution, respectively. After thorough vortexing (30 min), the mixtures were placed at room temperature, in the absence of light. The methanol solution (50%) was employed as the blank control and a standard curve was generated using Trolox solution. Absorbance values at 734 nm were determined and radical scavenging activities of ABTS and DPPH were calculated based on standard curve.

### 4.8. Data Statistics and Analysis

The results were reported as the mean ± standard deviation (SD) of three biological replicates. Statistical analysis was conducted by SPSS 24.0 (IBM Corporation, Armonk, NY, USA). To assess significant differences among the samples, a one-way analysis of variance (ANOVA) was employed, and the significance level was set at *p* < 0.05.

## 5. Conclusions

The present study showed that inhibiting spermidine degradation resulted in severe oxidative damage and a significant decrease in phenolic acid content in barley seedlings exposed to UV-B treatment. However, GABA application was found to alleviate the inhibition of phenolic acid biosynthesis by elevating the activities of key enzymes related to this process and thus ameliorated the adverse effects of UV-B stress by reducing increased electrolyte permeability and MDA content. In conclusion, spermidine mitigated oxidative damage and elevated accumulation of phenolic compounds in barley seedlings through degradation to GABA.

## Figures and Tables

**Table 1:** Content of phenolic acids (μg/g DW) in barley seedlings.

Phenolic Acid	Treatments	Phenolic Acids Content (μg/g DW)		
		Free	Bound	Total
Ferulic acid	CK	32.36 ± 1.40 ^b^	1817.78 ± 15.75 ^b^	1850.14 ± 14.35 ^b^
	A	24.76 ± 1.18 ^d^	1464.88 ± 3.73 ^c^	1489.64 ± 2.55 ^c^
	G	37.60 ± 1.50 ^a^	2363.78 ± 52.77 ^a^	2401.39 ± 54.27 ^a^
	E	23.63 ± 0.13 ^d^	1453.55 ± 47.84 ^c^	1477.18 ± 47.71 ^c^
	EG	28.00 ± 0.22 ^c^	1785.29 ± 26.96 ^b^	1813.29 ± 38.45 ^b^
*p*-Coumaric acid	CK	6.01 ± 0.01 ^a^	228.01 ± 2.74 ^b^	234.02 ± 2.73 ^b^
	A	3.93 ± 0.12 ^b^	146.96 ± 2.78 ^d^	150.89 ± 2.90 ^d^
	G	5.89 ± 0.08 ^a^	256.27 ± 3.01 ^a^	262.16 ± 2.93 ^a^
	E	3.75 ± 0.52 ^b^	172.69 ± 7.36 ^c^	176.44 ± 7.88 ^c^
	EG	4.21 ± 0.36 ^b^	190.64 ± 9.99 ^c^	194.84 ± 14.63 ^c^
Sinapic acid	CK	78.06 ± 1.71 ^b^	63.67 ± 0. 32 ^c^	141.73 ± 2.03 ^b^
	A	66.65 ± 0.26 ^d^	63.93 ± 0.11 ^c^	130.58 ± 0.15 ^d^
	G	86.03 ± 1.35 ^a^	69.40 ± 0.11 ^a^	155.43 ± 1.46 ^a^
	E	66.17 ± 0.07 ^d^	62.78 ± 0.49 ^d^	128.95 ± 0.43 ^d^
	EG	73.21 ± 0.10 ^c^	65.91 ± 0.38 ^b^	139.12 ± 0.69 ^c^
Caffeic acid	CK	38.25 ± 0.82 ^a^	2.24 ± 0.03 ^c^	40.49 ± 0.78 ^a^
	A	18.55 ± 0.24 ^d^	2.33 ± 0.01 ^bc^	20.88 ± 0.25 ^d^
	G	29.07 ± 2.44 ^b^	2.28 ± 0.08 ^b c^	31.34 ± 2.36 ^b^
	E	23.79 ± 0.13 ^c^	2.56 ± 0.23 ^ab^	26.34 ± 0.36 ^c^
	EG	29.03 ± 2.14 ^b^	2.73 ± 0.08 ^a^	31.76 ± 2.92 ^b^
Vanillic acid	CK	8.00 ± 0.24 ^b^	6.50 ± 0.12 ^a^	14.51 ± 0.50 ^b^
	A	4.49 ± 0.09 ^d^	5.24 ± 0.04 ^b^	9.74 ± 0.07 ^d^
	G	9.62 ± 0.15 ^a^	6.44 ± 0.06 ^a^	16.06 ± 0.13 ^a^
	E	4.50 ± 0.09 ^d^	4.80 ± 0.17 ^c^	9.31 ± 0.37 ^d^
	EG	6.27 ± 0.07 ^c^	4.99 ± 0.04 ^c^	11.25 ± 0.04 ^c^
*p*-Hydroxybenzoic acid	CK	4.43 ± 0.97 ^b^	6.76 ± 0.07 ^a^	11.19 ± 0.90 ^b^
	A	4.72 ± 0.20 ^b^	3.49 ± 0.11 ^b^	8.20 ± 0.31 ^c^
	G	7.87 ± 0.46 ^a^	7.74 ± 0.77 ^a^	15.61 ± 1.23 ^a^
	E	3.84 ± 0.06 ^b^	4.16 ± 0.22 ^b^	7.99 ± 0.16 ^c^
	EG	4.28 ± 0.10 ^b^	6.68 ± 0.36 ^a^	10.97 ± 0.38 ^b^
Protocatechuic acid	CK	4.23 ± 0.14 ^b^	1.35 ± 0.01 ^b^	5.58 ± 0.22 ^b^
	A	4.16 ± 0.37 ^b^	0.28 ± 0.02 ^c^	4.44 ± 0.55 ^b^
	G	12.37 ± 0.90 ^a^	3.43 ± 0.56 ^a^	15.80 ± 1.83 ^a^
	E	ND	1.00 ± 0.53 ^b^	1.00 ± 0.53 ^c^
	EG	5.36 ± 0.56 ^b^	1.30 ± 0.17 ^b^	6.66 ± 1.04 ^b^
Syringic acid	CK	2.50 ± 0.08 ^a^	4.38 ± 0.06 ^b^	6.87 ± 0.14 ^b^
	A	ND	3.82 ± 0.09 ^c^	3.82 ± 0.09 ^d^
	G	2.40 ± 0.01 ^a^	4.77 ± 0.01 ^a^	7.17 ± 0.01 ^a^
	E	ND	4.28 ± 0.01 ^b^	4.28 ± 0.01 ^c^
	EG	ND	4.01 ± 0.19 ^c^	4.01 ± 0.19 ^cd^

Different lowercase letters (a–d) represent significant difference of each phenolic acid content among different treatments (n = 3; *p* < 0.05).

**Table 2:** Free radical scavenging ability of ABTS and DPPH in barley seedlings.

Treatments	ABTS Values (μmol TE/g DW)			DPPH Values (μmol TE/g DW)		
	Free	Bound	Total	Free	Bound	Total
CK	39.80 ± 3.02 ^bc^	43.57 ± 2.96 ^a^	83.37 ± 3.25 ^a^	14.89 ± 2.09 ^bc^	25.01 ± 0.39 ^b^	39.90 ± 2.29 ^b^
A	48.20 ± 3.03 ^a^	35.61 ± 2.58 ^b^	83.80 ± 2.85 ^a^	14.52 ± 0.54 ^c^	21.27 ± 0.49 ^d^	35.79 ± 0.87 ^d^
G	41.17 ± 1.27 ^b^	36.37 ± 3.10 ^b^	77.54 ± 3.08 ^b^	17.87 ± 0.66 ^a^	25.66 ± 0.47 ^a^	43.53 ± 0.89 ^a^
E	37.04 ± 1.62 ^c^	44.13 ± 2.84 ^a^	81.18 ± 3.64 ^ab^	16.37 ± 0.47 ^ab^	23.55 ± 0.31 ^c^	39.91 ± 0.49 ^b^
EG	33.51 ± 3.18 ^d^	43.55 ± 1.49 ^a^	77.06 ± 4.06 ^b^	13.92 ± 1.16 ^c^	24.05 ± 0.24 ^c^	37.97 ± 1.14 ^c^

Different lowercase letters (a–d) represent significant difference of each phenolic acid content among different treatments (n = 3; *p* < 0.05).

## Data Availability

The data presented in this study are available on request from the corresponding author.
